# Metadata-driven identification of high-temperature superconductor candidates

**DOI:** 10.1038/s41598-025-29162-4

**Published:** 2025-12-29

**Authors:** Artur P. Durajski, Pawel Niegodajew, Izabela A. Wrona

**Affiliations:** 1 Faculty of Electrical Engineering, Czestochowa University of Technology, Armii Krajowej 17, Czestochowa, 42-200 Poland; 2 Department of Thermal Machinery, Czestochowa University of Technology, Armii Krajowej 21, Czestochowa, 42-200 Poland

**Keywords:** High-$T_c$ superconductivity, Quaternary hydrides, Electronegativity, Search strategy, Chemistry, Materials science, Physics

## Abstract

The recent discovery of high-$T_c$ superconductivity in hydrogen-rich materials has driven significant progress in superconductor research, particularly in binary and ternary hydrides. However, more complex hydrogen-rich systems remain largely unexplored. Our analysis suggests that while quaternary hydrides adhere to key design principles identified in simpler systems, their distribution within the relevant chemical space is notably sparse, underscoring the need for targeted investigations. By applying stringent selection criteria based on hydrogen fraction, mass ratio, and electronegativity, we systematically reduce the initial database of 38,517,336 possible quaternary hydrides to 1,060,019 promising candidates, representing just 2.75% of the total compositional space. This focused approach enhances the efficiency of computational screening and experimental validation by prioritizing materials with the highest likelihood of exhibiting superconductivity. The results provide a well-defined framework for the discovery of next-generation superconductors, guiding both theoretical exploration and experimental synthesis.

## Introduction

Superconductivity, the phenomenon where electrical resistance drops to zero below a critical temperature ($T_c$), has captivated researchers since its discovery in 1911^1^. Its potential for technological applications, such as lossless power transmission, ultra-sensitive magnetic sensors, and quantum computing, positions it as a cornerstone of modern science and engineering. However, practical applications have been limited by the low $T_c$ of early superconductors, which required cooling to near absolute zero. The discovery of high-temperature superconductors in the 1980s, particularly in copper-based oxides, revolutionized the field by achieving superconductivity at liquid nitrogen temperatures^2^. Despite these advances, achieving room-temperature superconductivity at ambient or moderate conditions remains the ultimate goal, with profound implications for energy efficiency and technological innovation.

Hydrogen-rich compounds have emerged as promising materials in this quest, offering a way to overcome these limitations. The journey into hydrogen-rich compounds as high-temperature superconductors began with the visionary hypothesis of Ashcroft^3^ who proposed that hydrogen under extremely high pressure could achieve metallization and exhibit superconductivity. While experimental limitations hindered the confirmation of this idea, Ashcroft’s later proposal in 2004 broadened the scope by introducing the concept of chemical “pre-compression” in hydrides^4^. This approach suggested that the superconducting properties of hydrogen could be accessed by combining it with heavier elements, allowing metallization at experimentally achievable conditions^4^. This hypothesis started a new era in the field of superconductivity, redirecting efforts toward hydrogen-based materials as promising candidates for high-temperature superconductors. Although early experimental attempts were only modestly successful, the idea laid the foundation for a new field of research that led to groundbreaking discoveries in the decades that followed.

Existing research on hydrogen-rich binary and ternary hydrides has revealed the remarkable superconducting properties of these materials, with critical temperatures ($T_c$) approaching or exceeding room temperature in several cases. Binary compounds such as $\hbox {H}_3$S ($T_c=203$ K)^5^ and $\hbox {LaH}_{10}$ ($T_c=250$ K)^6^ have shown exceptional performance, albeit at pressures exceeding 150 GPa. Subsequent exploration of ternary hydrides has expanded this search by introducing additional elements, enabling novel chemical configurations and opening pathways to high $T_c$ at more practical pressures. Notable systems include $\hbox {LaBeH}_8$ with a $T_c$ of 110 K at 80 GPa^7^ and $\hbox {LaB}_2\hbox {H}_8$ with a $T_c$ above 105 K at a pressure of 90 GPa (remains stable at pressures down to 59 GPa)^8^. In addition, compounds such as $\hbox {Mg}_2\hbox {IrH}_6$ ($T_c=160$ K) and Mg($\hbox {BH}_4$)$_2$ ($T_c=140$ K) demonstrate the potential for ambient pressure superconductivity^9,10^. These advances illustrate a critical shift towards the design of materials that combine high $T_c$ values with lower stabilization pressures.

Despite considerable progress, a comprehensive understanding of the physicochemical mechanisms underlying the phenomenon of superconductivity in these compounds remains largely overlooked in analyses. As a result, initial efforts to identify promising materials have been hampered by the lack of reliable empirical criteria. Many of these efforts relied on the presence of high-energy phonons or the observation of highly symmetric crystal structures and a high density of states (DOS) at the Fermi level^11–14^. Although these features were found to be necessary, they were ultimately insufficient to achieve high $T_c$. However, recent studies have revealed more nuanced relationships. For example, Belli *et al.*^15^ analyzed 178 binary hydride superconductors and found that $T_c$ is closely related to a network parameter derived from the hydrogen fraction and the cube root of the fraction of the DOS at the Fermi level contributed by hydrogen atoms. While this finding provided important insights into the role of hydrogen in the context of superconductivity, its reliance on computationally or experimentally intensive methods such as ab initio crystal structure prediction or X-ray diffraction limits its practicality for rapid screening of novel materials.

Building on advances in binary hydrides, Liu *et al.*^16^ contributed to the understanding of ternary hydrides by proposing five key approaches to enhance their superconducting properties. These strategies include the following: (1) optimizing covalent interactions to strengthen bonds, (2) increasing hydrogen content to form cage-like structures, (3) doping to reduce pressure requirements, (4) tuning electronic properties to enhance electron-phonon coupling, and (5) selecting pre-compression elements to aid stabilization. Although these approaches remain largely theoretical and require experimental validation, they provide a valuable framework for future research.

Recently, Wrona *et al.*^17^ extended this line of research by analyzing a broader database of over 296 binary hydrides (580 data points from 191 papers published between 2007 and 2023) and introducing a simpler yet effective metrics for predicting superconductivity potential: the ratio of the molecular mass of heavier atoms to hydrogen ($M_X/M_H$) and hydrogen fraction ($H_f$). Their study showed that compounds with lower $M_X/M_H$ ratios are more likely to have higher $T_c$ values, with a $28\%$ probability of identifying superconductors with $T_c$ above 200 K in the range $0< M_X/M_H < 15$. On the other hand, to obtain high-$T_c$, the $H_f$ coefficient must take values above 0.6. While these metrics do not guarantee the identification of high-temperature superconductors, they provide a pragmatic tool for narrowing down candidates in the early stages of material selection, potentially reducing experimental and computational requirements. Subsequently, Wrona *et al.*^18^ extended their investigation to ternary hydrogen-rich compounds, analyzing a dataset of over 560 compounds containing 1042 data points from 186 papers published between 2015 and 2024. Their results underscored the importance of compositional and structural parameters in predicting superconducting performance, identifying specific ranges of $M_X/M_H$, $H_f$, and electronegativity ($\chi$) that maximize the likelihood of achieving high $T_c$ values. In particular, compounds with $M_X/M_H\in (0,40)$, $H_f\in (0.8,1.0)$, and $\chi \in (2.0,2.1)$ exhibited a remarkably high probability of manifesting $T_c$ above 200 K, with some exceeding 300 K. These results highlight the multidimensional nature of superconducting material optimization, where a balance between electronegativity, enhanced electron-phonon coupling, and hydrogen-rich environments together enable the achievement of high critical temperatures at manageable pressures.

The studies of Wrona *et al.*^17,18^ represent the most comprehensive statistical analyses of hydrogen-rich superconductors to date, providing a solid foundation for exploring more complex systems such as quaternary hydrides. These materials introduce additional compositional complexity, which may enable fine-tuning of electronic structure, phonon interactions, and stability at reduced pressures. However, this complexity also presents new challenges in predicting and synthesizing stable, high-performance superconductors. In this work, we systematically evaluate whether quaternary hydrides can push the boundaries of high-$T_c$ superconductivity by leveraging statistical insights, trends, and theoretical forecasting. By extending predictive frameworks developed for simpler hydrogen-rich systems, we seek to identify physicochemical parameters for promising candidates that could bring us closer to realizing practical room-temperature superconductors at accessible pressures. Finally, based on our analysis, we identify a statistically defined subset of quaternary hydrides with the highest likelihood of exhibiting superconductivity, providing a well-defined starting point for future computational and experimental exploration.

## Brief state of the art on binary and ternary hydride superconductors

**Fig. 1:**
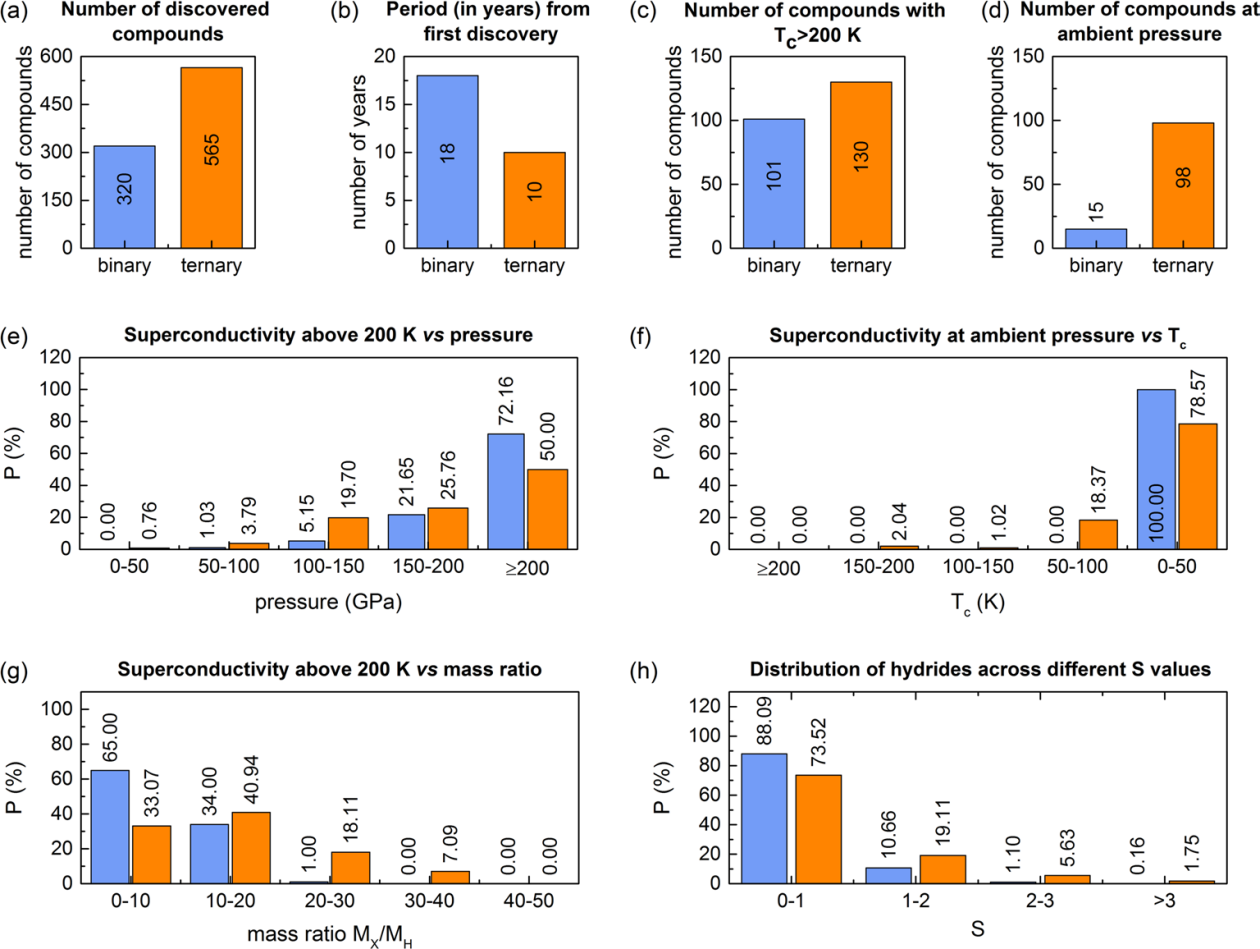
Comparison of binary (blue bars) and ternary hydrides (orange bars). (**a**) The number of discovered binary and ternary hydrides. (**b**) The period (in years) between the first discovery and 2024. (**c**) The number of binary and ternary hydrides (considering different pressures) that exhibit superconductivity above 200 K. (**d**) The number of binary and ternary hydrides that remain stable and exhibit superconductivity at ambient pressure (0 GPa). (**e**) Probability distribution of superconductors with $T_c$ above 200 K *vs* pressure. (**f**) Probability distribution of superconductors at ambient pressure *vs* critical temperature. (**g**) Probability distribution of superconductors with $T_c$ above 200 K *vs* mass ratio. (**h**) The distribution of binary and ternary hydrides across different *S* values.

Hydrogen-rich compounds have been at the forefront of the search for high-temperature superconductors, with binary and ternary hydrides playing a central role. While early studies primarily focused on binary hydrides, recent advancements have demonstrated that incorporating a third element can enhance stability, reduce synthesis pressures, and improve superconducting properties. Understanding the trends and limitations of these systems is essential not only for evaluating their potential but also for guiding the exploration of more complex materials, such as quaternary hydrides. To this end, we analyze key statistical trends that distinguish binary and ternary hydrides, as presented in Fig. 1, to assess their impact on superconducting performance and identify strategies that could be extended to quaternary systems. To conduct this comprehensive analysis, we have drawn upon extensive databases collected in recent studies, including the seminal works by Wrona *et al.*^17^ and^18^, which have been systematically expanded with the latest research findings from 2024^19–34^.

A fundamental aspect of this landscape is the number of discovered superconducting compounds. As shown in Fig. 1a, ternary hydrides significantly outnumber their binary counterparts. To date, 320 unique binary hydrides have been identified, whereas ternary compounds have surged ahead, with 565 unique superconducting phases reported. Moreover, as highlighted in Fig. 1b, the much faster rate of discovery of ternary hydrides, despite their shorter research history, underscores their growing importance, reflecting advancements in high-pressure synthesis techniques and computational material discovery.

Beyond their sheer abundance, the superconducting performance of these systems is of paramount interest. Figure 1c compares the number of binary and ternary hydrides exhibiting superconductivity with $T_c > 200$ K. In this analysis, we consider not only the unique chemical compositions of hydrides but also the various pressure conditions under which superconductivity is observed. Consequently, the dataset extends to 665 data points for binary hydrides and 1057 data points for ternary hydrides. While the number of such compounds with $T_c > 200$ K is only slightly greater for ternary hydrides (130) than for binary ones (101), a far more striking difference emerges in their ability to sustain superconductivity at ambient pressure. Figure 1d highlights a notable disparity between binary and ternary hydrides regarding their stability at ambient pressure (0 GPa). Only 15 binary hydrides exhibit superconductivity under this condition, whereas ternary systems vastly outperform them, with 98 known ambient-pressure superconductors–a six-fold increase. These statistics demonstrate that the presence of a third element likely plays a pivotal role in stabilizing their structures, thereby reducing the necessity for extreme external pressure to maintain the superconducting phase.

The more detailed interplay between high-temperature superconductivity and pressure is further examined in Fig. 1e, which presents the probability distribution of superconductors with $T_c > 200$ K as a function of pressure. The data reveal a notable distinction: at lower pressures (below 150 GPa), ternary hydrides dominate, whereas at extreme pressures (above 200 GPa), binary hydrides are more prevalent. Moreover, this trend hints at the existence of distinct stabilization mechanisms in ternary systems, such as chemical pre-compression or optimized bonding configurations, which could be crucial for understanding and designing future high-$T_c$ materials.

Further reinforcing the importance of ambient-pressure superconductivity, Fig. 1f categorizes superconducting hydrides that remain stable at ambient pressure by their critical temperature $T_c$. A stark contrast between the binary and ternary systems is apparent: all binary hydrides that persist at ambient pressure exhibit low values of $T_c$ (below 50 K), while ternary hydrides dominate the high-temperature range, including compounds exceeding 150 K. The absence of binary superconductors with $T_c > 50$ K at zero pressure reinforces the notion that binary systems generally require extreme environments to achieve high-$T_c$ superconductivity.

Moving beyond pressure-related trends, Fig. 1g examines the correlation between superconductivity and the mass ratio of non-hydrogen elements to hydrogen ($M_X/M_H$) - a parameter identified as an important factor in compounds exhibiting high-$T_c$ superconductivity^17,18^. The results indicate that lighter compositions (mass ratio 0–10) strongly favor binary systems, with a probability of approximately $65\%$, whereas ternary hydrides exhibit a more balanced distribution across the 0–10 and 10–20 mass ratio ranges. This trend suggests that lighter atomic masses are generally more favorable for achieving high-$T_c$ superconductivity, as expected from theoretical predictions emphasizing the role of hydrogen-rich structures in sustaining strong electron-phonon interactions. However, a key distinction emerges between binary and ternary systems: while binary hydrides exhibit a greater restriction on achieving $T_c > 200$ K - predominantly confined to the lowest mass ratio ranges ($0-10$ and $10-20$) - ternary hydrides display a nonzero probability of reaching high $T_c$ values even at larger mass ratios. Notably, in the 20–30 range, ternary hydrides still maintain a visible presence, whereas binary systems are nearly absent. The trend continues in the 30–40 range, where the probability is significantly reduced but remains nonzero for ternary hydrides, in contrast to the complete absence of binary superconductors with $T_c > 200$ K. These results highlight that ternary hydrides are slightly less constrained by mass ratio limits, demonstrating a higher probability of high-$T_c$ superconductivity compared to binary hydrides, even as they approach the critical limit of 40, above which the probability drops to zero.

A more holistic superconducting performance metric is introduced in Fig. 1h, which presents the distribution of hydrides across different *S* values, a figure of merit proposed by Pickard *et al.*^13^. The *S* parameter balances high critical temperature and pressure constraints, offering a more comprehensive perspective on superconducting potential:

1 $$\begin{aligned} S=\frac{T_c}{\sqrt{T^2_c ({\textrm{MgB}}_2) + p^2}}, \end{aligned}$$

where $T_c$($\hbox {MgB}_2$) represents the critical temperature of $\hbox {MgB}_2$ (39 K) under ambient condition. Superconductors that exhibit higher values of *S* are considered to have superior performance, balancing high $T_c$ with manageable pressure requirements. The pressure dependence of $T_c$ for superconducting binary, ternary, and quaternary hydrides, along with the figure of merit *S* that quantifies the significance of each superconductor, is presented in the Supplementary Information (see Fig. S1). The data reveals that for the lowest *S* range ($0-1$), binary hydrides dominate ($\sim 90\%$), while ternary hydrides account for approximately $75\%$. However, for higher *S* values (1–2 and beyond), ternary hydrides become increasingly prevalent. Notably, at $S > 2$, a significant fraction of ternary compounds emerge, suggesting that multi-component hydrides may offer superior superconducting performance by balancing high $T_c$ with lower pressure requirements. This suggests that ternary compounds are more likely to achieve high-$T_c$ superconductivity under experimentally accessible conditions (see the Supplementary Information).

The observed trends, as illustrated in Fig. 1, suggest a crucial role for ternary hydrides in the advancement of superconductors, particularly in the stabilization of high-$T_c$ materials at lower pressures. This prompts a fundamental question: Can quaternary hydrides further push these boundaries? If so, what strategies should guide their study, and what specific parameters define the most promising compounds? More importantly, how can their exploration be accelerated by filtering out less promising candidates?

Building on the insights gained from binary and ternary hydrides, one can expect quaternary hydrides to continue the observed trend of improved superconducting performance. The statistical analysis presented above highlights key advantages of ternary hydrides over binary systems, including their higher abundance, increased stability at lower pressures, and broader range of favorable mass ratios, all of which contribute to enhanced superconducting potential. Given that ternary hydrides have already demonstrated a significant increase in the number of high-$T_c$ superconductors, particularly under ambient and moderate pressures, it is reasonable to anticipate that quaternary hydrides may further extend these benefits. By introducing an additional compositional degree of freedom, quaternary systems could enable even greater structural stability, reduced pressure requirements, and a wider parameter space for optimizing superconducting properties. The following section delves into this analysis, outlining the key factors that may govern quaternary hydrides and providing a framework for their targeted discovery.

## Towards quaternary hydrides

**Fig. 2:**
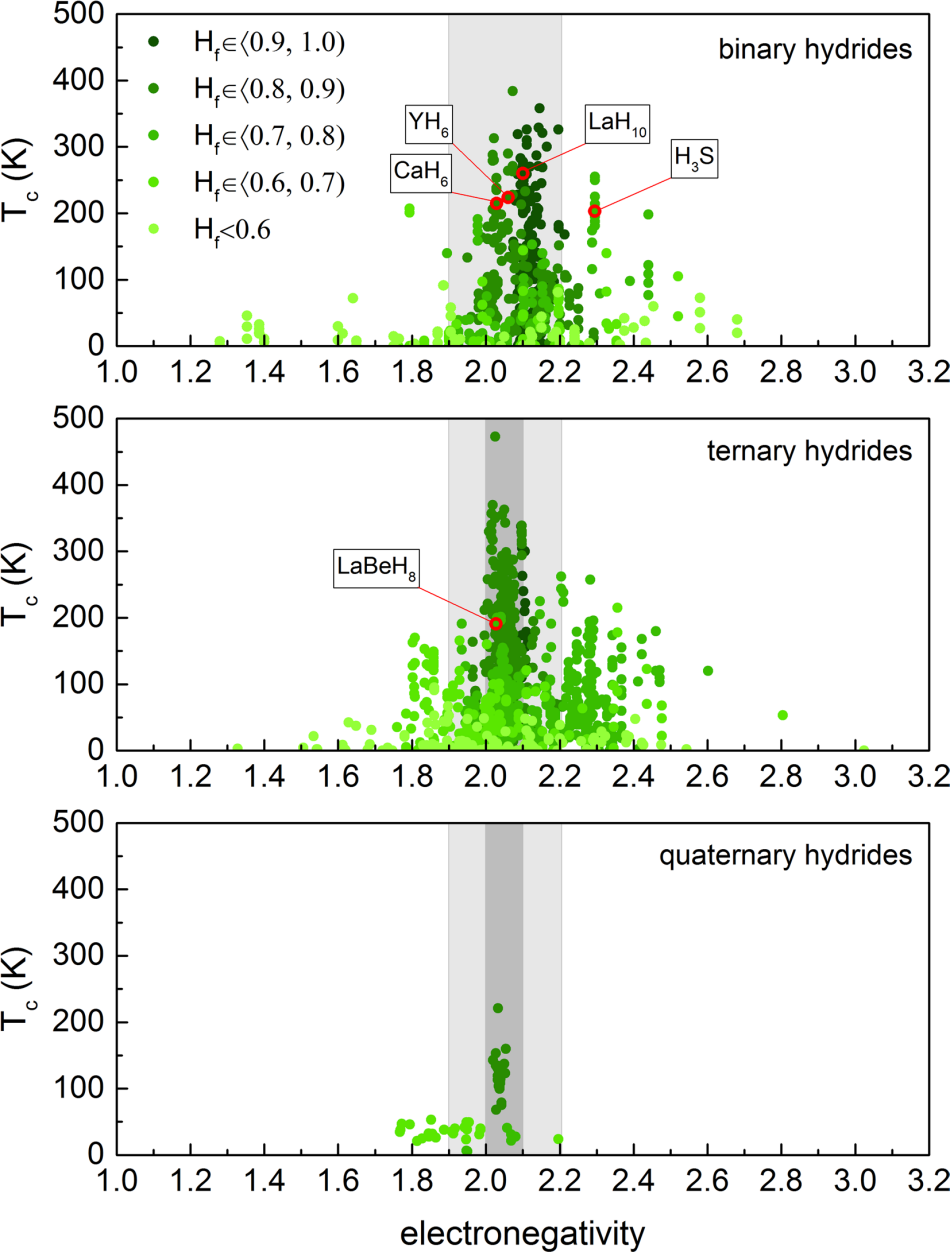
Electronegativity *vs* $T_c$ in hydrides: The graph illustrates how electronegativity affects the superconducting transition temperature ($T_c$) in binary, ternary, and quaternary hydrides, with points color-coded by hydrogen fraction ($H_f$). The data for binary and ternary hydrides are based on the databases from^17,18^, extended with the latest results^19–34^, while the data for quaternary hydrides are taken from^24,35–39^. Red circles mark the well-known, experimentally verified systems. The light gray area denotes the 1.9–2.2 electronegativity window derived from binary hydrides, while the darker gray marks a narrower 2.0–2.1 range reflecting the more compact clustering of high-$T_c$ ternary compositions.

Having established key statistical trends in binary and ternary hydrides, we now turn our attention to the next logical step in high-$T_c$ superconductor research: quaternary hydrides. These compounds introduce additional degrees of freedom in chemical composition and structural tuning, which could enable further optimization of superconducting properties. While their investigation remains in its early stages, quaternary hydrides hold the potential to refine key parameters such as hydrogen content, electronic structure, and phonon interactions - critical factors in achieving record-breaking critical temperatures. Our previous studies on ternary hydrides have established a robust correlation between electronegativity and superconducting properties^18^. In this work, we expand this analysis to encompass binary and quaternary hydrides, enabling a comprehensive comparison across varying levels of compositional complexity. To support this investigation, we have constructed a dedicated database of quaternary hydrides, comprising 49 compounds and 57 data points curated from six publications spanning 2022 to 2024^24,35–39^. This analysis seeks to determine whether the trends identified in simpler systems persist in quaternary compositions and to develop a systematic framework for identifying the most promising superconducting candidates.

Figure 2 illustrates the correlation between electronegativity and critical temperature ($T_c$) for binary, ternary, and quaternary hydride superconductors, with data points color-coded according to their hydrogen fraction ($H_f$). The electronegativity of multicomponent hydride compounds is determined using a weighted average of the electronegativities of their constituent elements:

2 $$\begin{aligned} \chi = \frac{\sum _{i=1}^{\eta } n_{\textrm{X}_i} \cdot \chi _{\textrm{X}_i} + n_{\textrm{H}} \cdot \chi _{\textrm{H}}}{\sum _{i=1}^{\eta } n_{\textrm{X}_i} + n_{\textrm{H}}}, \end{aligned}$$

where $\chi _{\textrm{X}_i}$ and $\chi _{\textrm{H}}$ represent the electronegativities (Pauling scale) of the non-hydrogen elements and hydrogen, respectively, while $n_{\textrm{X}_i}$ and $n_{\textrm{H}}$ denote the number of atoms of each element in the compound. This formulation extends across binary ($\eta =1$), ternary ($\eta =2$), and quaternary ($\eta =3$) hydrides, as well as even more complex systems.

Another key parameter in this analysis is the hydrogen fraction, defined as:

3 $$\begin{aligned} H_{f}=\frac{n_{\textrm{H}}}{\sum _{i=1}^{\eta } n_{\textrm{X}_i}+n_{\textrm{H}}}, \end{aligned}$$

where $H_f$ quantifies the relative abundance of hydrogen in the compound. Previous studies have shown that high $H_f$ values generally correlate with strong electron-phonon coupling, a crucial mechanism for high-$T_c$ superconductivity.

The analysis of results presented in Fig. 2 reveals distinct trends across these three classes of hydrides. Binary hydrides exhibit the highest $T_c$ values (approaching 400 K) predominantly within the electronegativity range of 1.9–2.2 and for materials with $H_f > 0.8$. A similar pattern emerges for ternary hydrides, where maximum $T_c$ values (reaching up to approximately 450 K) are observed within the same optimal electronegativity window. Although the dataset for quaternary hydrides is less extensive (limited to just 57 data points^24,35–39^), the observed trends are consistent with those identified for binary and ternary systems. The highest recorded $T_c$ in this class reaches approximately 220 K, with data points aligning well with the optimal electronegativity window established for simpler hydrides. This coherence suggests that the same fundamental mechanisms underlying high-$T_c$ superconductivity may extend to quaternary compositions. While further studies are needed to confirm these relationships, the emerging patterns reinforce the broader principles governing the design and discovery of superconducting hydrides. A crucial insight from this comparison is the importance of $H_f$. Across all three systems, higher $H_f$ values ($H_f \ge 0.8$) (represented by darker green points) consistently correlate with higher critical temperatures, particularly within the electronegativity window of 1.9–2.2 highlighted in gray.

Moreover, building on the trends observed in binary and ternary hydrides (see Fig. 1g), quaternary hydrides are expected to follow a similar progression in terms of mass ratio constraints for high-$T_c$ superconductivity. The mass ratio of heavier atoms to hydrogen is defined as:

4 $$\begin{aligned} M_{\textrm{X}}/{M_{\textrm{H}}}= \frac{\sum _{i=1}^{\eta } n_{\textrm{X}_i}\cdot M_{\textrm{X}_i}}{n_{\textrm{H}} \cdot M_{\textrm{H}}}, \end{aligned}$$

where $M_{\textrm{X}_i}$, and $M_H$ denote the molar masses of each element in the compound. While binary hydrides exhibit high-$T_c$ behavior predominantly for mass ratios up to 20, and ternary hydrides extend this threshold to approximately 40. This pattern suggests that as chemical complexity increases, the upper limit of favorable mass ratios shifts to higher values, continuing the trend observed in simpler hydrides. Therefore, it is reasonable to expect that the range for quaternary hydrides may extend slightly further, encompassing mass ratios beyond 40 while still maintaining the possibility of high-$T_c$ superconductivity.

The analysis presented here establishes a framework for identifying promising high-$T_c$ candidates among quaternary hydrides. By delineating optimal ranges for electronegativity, hydrogen fraction, and mass ratio, we provide a systematic approach for distinguishing compositions with the highest probability of exhibiting superconductivity at elevated temperatures. These criteria are applied in the next chapter to extract materials among quaternary hydrides that should be considered as potential candidates for high-$T_c$ superconductivity. By systematically filtering compositions based on the identified parameter ranges, the following analysis aims to highlight the most promising structures for further theoretical and experimental exploration.

## Statistical procedure and algorithmic screening of quaternary hydrides

To systematically explore the vast chemical space of quaternary hydrides and identify potential high-$T_c$ candidates, we implemented a combinatorial algorithm that integrates elemental metadata with descriptor-based statistical filtering. The procedure begins with an initial elemental selection step: only elements with well-defined electronegativity values and stable, non-radioactive isotopes were included, while noble gases were excluded due to their negligible tendency to form hydrides (87 non-hydrogen elements; see Table S7 in Supplementary Information). Hydrogen was treated as the essential light element, present in all compositions. For each unique triplet of heavier elements A, B, and C, stoichiometric coefficients were sampled from discrete sets ($\alpha , \beta , \gamma \in \{1,2,3\}$ and $\delta \in \{2,4,6,8,9,10,12,16,20,24,28,32,36\}$), generating a library of candidate compounds with general formulas $\hbox {A}_{\alpha }\hbox {B}_{\beta }\hbox {C}_{\gamma }\hbox {H}_{\delta }$. These limits were chosen to ensure chemically realistic stoichiometries and to maintain a manageable combinatorial size of the search space. They also reflect the stoichiometric diversity observed in known quaternary hydrides, such as $\hbox {LaSrB}_2\hbox {H}_{16}$^37^, $\hbox {ScY}_2\hbox {CaH}_{12}$^35^, $\hbox {K}_2\hbox {InCuH}_6$^24^, supporting the physical plausibility of the sampled range. Although extremely hydrogen-rich compositions may exist, they are frequently associated with structural instability or extreme synthesis conditions, and thus fall outside the scope of this initial metadata-based screening.

For each generated composition, three key descriptors were computed: the average electronegativity ($\chi$), the hydrogen fraction ($H_f$), and the mass ratio of non-hydrogen atoms to hydrogen ($M_X/M_H$). These quantities were evaluated using established definitions (Eqs. 2–4) and were directly linked to the statistical likelihood of achieving high superconducting transition temperatures, as identified in previous works on binary and ternary systems^17,18^. The algorithm then applied a threefold filtering criterion, retaining only those compounds that satisfied $\chi \in [2.0,2.1]$, $H_f \ge 0.8$, and $M_X/M_H \le 20$. The chosen $\chi$ interval was informed by the compact clustering of high-$T_c$ ternary compositions observed in Fig. 2, whereas the $M_X/M_H$ threshold reflects that both binary and ternary hydrides exhibit high-$T_c$ behavior predominantly for mass ratios up to 20 (see Fig. 1g). This domain of parameters corresponds to the region most strongly correlated with achieving $T_c > 200$ K in binary and ternary superconductors. The influence of individual descriptor values on the total number of generated compounds is presented in the Supplementary Information. Moreover, list of quaternary hydrides reported in the literature that meet $H_f$ and $\chi$ criteria is also provided in Supplementary Information.

The total chemical space of quaternary hydrides defined by the considered $\alpha$, $\beta$, $\gamma$, and $\delta$ values comprises 38,517,336 unique compositions when no restrictions are applied. The statistical filtering procedure reduced this set to 1,060,019 candidates (only 2.75% of the original space) representing the most promising subset for further investigation. The resulting database was cross-referenced with available experimental and theoretical literature to distinguish previously reported hydrides from unexplored combinations. The final output thus provides a curated list of statistically favorable quaternary compositions, serving as a foundation for subsequent first-principles and experimental studies.

To facilitate verification and further exploration, the resulting dataset of 1,060,019 screened quaternary hydrides is publicly available^40^. Beyond narrowing the search space, this approach enhances the efficiency of computational screening and experimental validation by prioritizing materials with the highest statistical likelihood of superconductivity, thereby streamlining the discovery process for next-generation hydrogen-rich superconductors.

## Summary

This study represents the first systematic attempt to narrow the vast chemical space of quaternary hydrides through a comprehensive meta-analysis of binary, ternary, and quaternary systems. Building on the observed trends, we anticipate that quaternary hydrides will extend the advantages of ternary systems, offering improved superconducting performance, enhanced stability at lower pressures, and a broader range of favorable compositions. By identifying key design parameters, hydrogen fraction $H_f \ge 0.8$, mass ratio $M_X/M_H \le 20$, and electronegativity $\chi \in [2.0,2.1]$, we establish a targeted selection strategy that reduces the number of viable candidates to just $2.75\%$ of the total compounds generated under stoichiometric constraints. This refined approach not only streamlines the search for high-$T_c$ superconductors but also enhances the feasibility of computational modeling and experimental validation. The resulting database provides a well-defined starting point for both theoretical studies and synthesis efforts, accelerating the discovery of quaternary hydrides with practical superconducting applications.

## Supplementary Information

Supplementary Information.

## Data Availability

The database containing 1,060,019 potential quaternary hydride superconductors is available on the Zenodo public database under accession code 17523714.
