# Supplementary material for: Metadata-driven identification of high-temperature superconductor candidates
Source: Sci Rep. 2025 Dec 29;15:44858. doi: 10.1038/s41598-025-29162-4 (PMC12749985; doi:10.1038/s41598-025-29162-4)
Supplement: Supplementary file 1 — Supplementary Information. [file 41598_2025_29162_MOESM1_ESM.pdf]

Supplementary Information for

Metadata-driven identification of high-temperature superconductor candidates

Artur P. Durajski,[1,*] Pawel Niegodajew,[2] and Izabela A. Wrona[1]

[1] *Faculty of Electrical Engineering, Czestochowa University of Technology, Armii Krajowej 17, Czestochowa, 42-200, Poland*[*]

[2] *Department of Thermal Machinery, Czestochowa University of Technology, Armii Krajowej 21, 42-200 Czestochowa, Poland*

(Dated: November 11, 2025)

Contents

1. Pressure dependence of T_c values for superconducting hydrides 2
2. Binary, ternary, and quaternary hydrides at ambient pressure 2
3. List of quaternary hydrides reported in the literature with $H_f \geq 0.8$ 6
4. Filtering criteria vs total number of generated compounds 6
5. Electronegativity values (Pauling scale) of elements used to create hydrogen compounds 7

References 7

* artur.durajski@pcz.pl

1. Pressure dependence of T_c values for superconducting hydrides

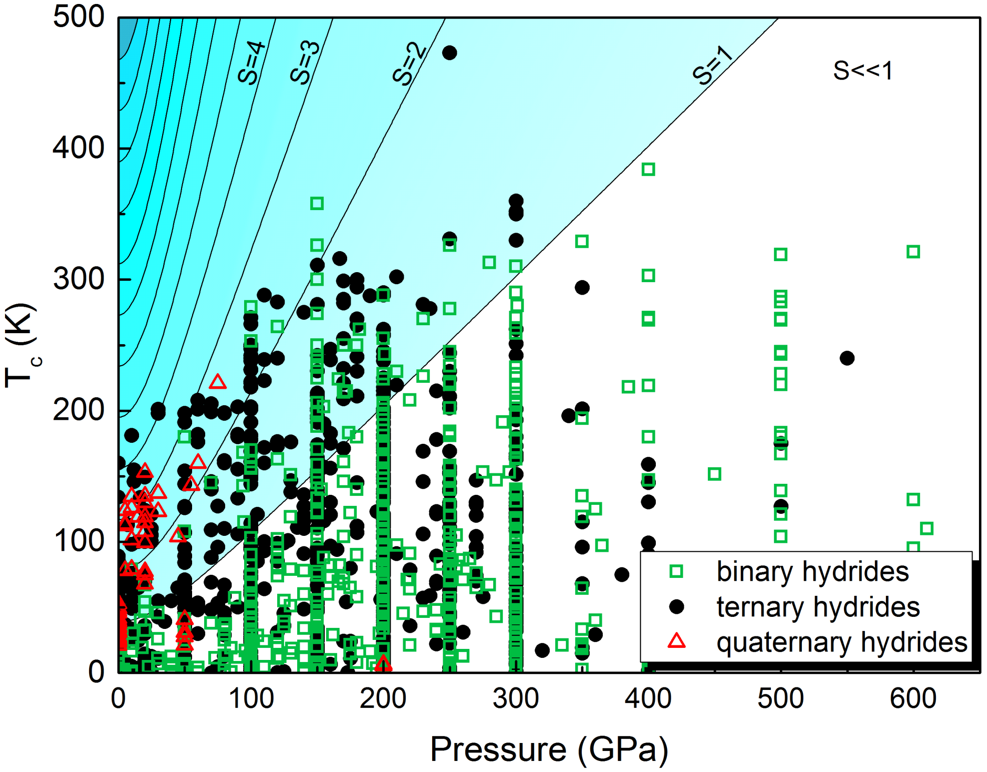

FIG. S1. Pressure dependence of T_c for binary (green squares), ternary (black circles) and quaternary (red triangles) hydrides, with shaded regions indicating ranges of S [1]. The results for binary and ternary hydrides are taken from Ref. [2, 3].

2. Binary, ternary, and quaternary hydrides at ambient pressure

TABLE S1. List of binary hydrides at ambient pressure.

**Name**	H_f	M_X/M_H	χ	T_c **(K)**	**Ref.**
CrH	0.50	51.58	1.93	10.60	10.1038/srep17764
NbH_2	0.67	46.08	2.00	1.50	10.1063/1.4866179
NbH_2	0.67	46.08	2.00	1.84	10.1016/j.arabjc.2022.104347
PdH_2	0.67	52.79	2.20	24.00	10.1016/j.ijhydene.2022.12.312
TaH_2	0.67	89.76	1.97	1.53	10.1016/j.arabjc.2022.104347
Th_2H_7	0.78	65.77	2.00	0.37	10.1016/j.ssc.2020.113820
ThH_2	0.67	115.10	1.90	0.02	10.1016/j.ssc.2020.113820
ThH_3	0.75	76.73	1.98	6.10	10.1016/j.ssc.2020.113820
TiH_2	0.67	23.74	1.98	0.00	10.1038/srep28102
TiH_2	0.67	23.74	1.98	7.00	10.1038/srep28102
VH_2	0.67	25.27	2.01	0.50	10.1063/1.4866179
ZrH_3	0.75	30.17	1.98	11.70	10.1016/j.commatsci.2017.03.021

TABLE S2. List of ternary hydrides at ambient pressure.

**Name**	H_f	M_X/M_H	χ	T_c **(K)**	**Ref.**
$AgMg_3H$	0.20	179.35	1.61	8.00	10.1103/PhysRevLett.132.166001
Al_2B_2H	0.20	74.98	1.90	52.64	10.1103/PhysRevMaterials.7.114802
Al_2MnH_6	0.67	18.01	2.00	43.90	10.1002/adfm.202404043
Al_2MnH_6	0.67	18.01	2.00	66.00	10.1016/j.mtphys.2024.101374
Al_2ReH_6	0.67	39.71	2.04	36.40	10.1002/adfm.202404043
Al_2TcH_6	0.67	25.13	2.04	43.10	10.1002/adfm.202404043
$AlHgH_3$	0.60	75.26	2.04	28.40	10.1002/adfm.202404043
Au_2NaH_6	0.67	68.94	2.13	89.00	10.1103/PhysRevLett.132.166001
$AuNa_3H$	0.20	263.83	1.51	4.00	10.1103/PhysRevLett.132.166001
$BaMgH_3$	0.60	53.45	1.76	36.00	10.1103/PhysRevLett.132.166001
Ca_2AgH_6	0.67	31.09	1.90	35.90	10.1002/adfm.202404043
Ca_2LiH_6	0.67	14.40	1.80	33.00	10.1002/adfm.202404043
Ca_2PdH_6	0.67	30.85	1.93	26.00	10.1016/j.mtphys.2024.101374
Ca_2PtH_6	0.67	45.51	1.94	20.00	10.1016/j.mtphys.2024.101374
CaB_3H	0.20	71.93	1.86	39.30	10.1088/0256-307X/40/1/017402
CdN_2H_8	0.73	17.41	2.31	0.20	10.1063/5.0127365
$CeSiH_7$	0.78	23.84	2.05	20.70	10.1103/PhysRevB.110.024513
Ga_2MnH_6	0.67	32.14	2.04	43.00	10.1016/j.mtphys.2024.101374
Ga_2OsH_6	0.67	54.51	2.11	20.90	10.1002/adfm.202404043
Ga_2RuH_6	0.67	39.77	2.11	26.90	10.1002/adfm.202404043
Ga_2RuH_6	0.67	39.77	2.11	16.00	10.1016/j.mtphys.2024.101374
Hf_3AlH	0.20	557.99	1.54	2.00	10.1103/PhysRevLett.132.166001
$HgHf_3H$	0.20	730.22	1.62	1.00	10.1103/PhysRevLett.132.166001
HgN_2H_8	0.73	28.35	2.33	0.01	10.1063/5.0127365
In_2MnH_6	0.67	47.05	2.03	45.00	10.1016/j.mtphys.2024.101374
$IrAl_3H$	0.20	271.00	1.85	27.00	10.1103/PhysRevLett.132.166001
IrW_3H	0.20	737.84	2.30	4.35	10.1103/PhysRevLett.132.166001
$KAlH_3$	0.60	21.85	1.81	52.00	10.1088/2752-5724/ad4a94
$KCdH_3$	0.60	50.10	1.82	12.30	10.1002/adma.202307085
$KInH_3$	0.60	50.90	1.84	72.90	10.1002/adfm.202404043
$LaSiH_7$	0.78	23.67	2.04	41.20	10.1103/PhysRevB.110.024513
$LaYH_2$	0.50	113.00	1.68	0.47	10.1088/1674-1056/ad41b8
$LaYH_3$	0.60	75.34	1.78	0.00	10.1088/1674-1056/ad41b8
Li_2AuH_2	0.40	104.59	1.78	20.80	10.1002/adfm.202404043
Li_2AuH_6	0.67	34.86	1.97	54.00	10.1016/j.mtphys.2024.101374
Li_2CuH_6	0.67	12.80	1.90	86.00	10.1002/adfm.202404043
Li_2CuH_6	0.67	12.80	1.90	80.00	10.1016/j.mtphys.2024.101374
Li_4BeH_5	0.50	7.30	1.65	37.20	10.1002/adfm.202404043
$LiPdH_2$	0.50	56.23	1.90	43.20	10.1002/adma.202307085
Lu_2NH_2	0.40	180.53	2.00	0.20	10.1063/5.0151844
Lu_2NH_3	0.50	120.35	2.03	10.50	10.1038/s41467-023-41005-2
$Lu_3N_2H_3$	0.38	182.85	2.06	4.90	10.1038/s41467-023-41005-2
Lu_4NH_3	0.38	236.07	1.84	8.10	10.1063/5.0215212
$LuNH$	0.33	187.48	2.17	16.00	10.1038/s41467-023-41005-2
Mg_2B_2H	0.20	69.67	1.78	23.25	10.1103/PhysRevMaterials.7.114802
Mg_2CoH_6	0.67	17.78	1.97	30.00	10.1016/j.mtphys.2024.101374
Mg_2IrH_6	0.67	39.82	2.00	160.00	10.1103/PhysRevLett.132.166001
Mg_2IrH_6	0.67	39.82	2.00	77.00	10.1038/s41524-024-01214-9
Mg_2IrH_6	0.67	39.82	2.00	65.00	10.1016/j.mtphys.2024.101374
Mg_2NiH_6	0.67	17.74	1.97	22.70	10.1002/adfm.202404043
Mg_2PdH_6	0.67	25.63	2.00	66.50	10.1038/s41524-024-01214-9

Mg_2PtH_6	0.67	40.29	2.01	80.40	10.1038/s41524-024-01214-9
Mg_2PtH_6	0.67	40.29	2.01	73.00	10.1016/j.mtphys.2024.101374
Mg_2RhH_6	0.67	25.05	2.01	48.50	10.1038/s41524-024-01214-9
Mg_2RhH_6	0.67	25.05	2.01	59.00	10.1016/j.mtphys.2024.101374
MgB_2H	0.25	45.56	1.90	67.00	10.1103/PhysRevLett.123.077001
$MgCu_3H$	0.20	213.24	1.84	42.00	10.1002/adfm.202304919
Mo_2B_2H	0.20	211.83	2.12	21.54	10.1103/PhysRevMaterials.7.114802
MoH_4N	0.80	19.93	2.29	103.00	10.1103/PhysRevLett.131.076001
$NaAlH_3$	0.60	23.68	1.79	54.00	10.1088/2752-5724/ad4a94
$NaCdH_3$	0.60	52.09	1.80	14.00	10.1002/adma.202307085
$NaInH_3$	0.60	52.89	1.82	85.90	10.1002/adfm.202404043
$Ni_2H_3B_2$	0.33	38.73	1.98	101.00	10.1103/PhysRevB.107.174512
Pd_3NaH	0.20	318.73	1.67	3.00	10.1103/PhysRevLett.132.166001
PdH_4N	0.80	26.08	2.14	30.00	10.1103/PhysRevLett.131.076001
Pt_3NaH	0.20	431.46	1.74	1.00	10.1103/PhysRevLett.132.166001
Pt_3ScH	0.20	458.66	1.78	1.00	10.1103/PhysRevLett.132.166001
Rh_3MgH	0.20	276.94	1.85	8.00	10.1103/PhysRevLett.132.166001
Ru_3MgH	0.20	261.42	1.83	11.00	10.1103/PhysRevLett.132.166001
$ScAlH_6$	0.67	18.01	1.90	37.00	10.1002/adfm.202404043
$ScCaH_6$	0.67	26.23	1.85	74.00	10.1002/adfm.202404043
$ScLuH_2$	0.50	123.36	1.68	0.16	10.1088/1674-1056/ad41b8
$ScLuH_3$	0.60	82.19	1.78	0.00	10.1088/1674-1056/ad41b8
$ScYH_2$	0.50	95.43	1.68	0.20	10.1088/1674-1056/ad41b8
$ScYH_3$	0.60	63.69	1.78	0.00	10.1088/1674-1056/ad41b8
Sr_2NiH_6	0.67	30.21	1.93	25.00	10.1016/j.mtphys.2024.101374
SrB_3H	0.20	96.45	1.82	47.20	10.1088/0256-307X/40/1/017402
$SrGaH_6$	0.67	28.03	1.95	25.20	10.1002/adfm.202404043
Ta_3NiH	0.20	569.87	2.02	13.00	10.1103/PhysRevLett.132.166001
Ti_2B_2H	0.20	103.11	1.69	43.27	10.1103/PhysRevMaterials.7.114802
$TiCuH_6$	0.75	20.60	1.96	81.00	10.1038/s41524-024-01214-9
W_3ScH	0.20	698.75	1.97	6.00	10.1103/PhysRevLett.132.166001
Y_2AgH_6	0.67	33.60	1.90	38.80	10.1002/adfm.202404043
Y_2LiH_6	0.67	16.91	1.80	45.00	10.1002/adfm.202404043
Y_2PdH_6	0.67	33.36	1.93	28.00	10.1016/j.mtphys.2024.101374
Y_2PtH_6	0.67	48.02	1.94	20.00	10.1016/j.mtphys.2024.101374
$YAlH_6$	0.67	19.99	1.90	36.00	10.1002/adfm.202404043
YB_3H	0.20	87.59	1.84	34.00	10.1088/0256-307X/40/1/017402
$YHgH_3$	0.60	63.93	2.01	34.00	10.1002/adfm.202404043
YH_2N	0.33	41.31	2.17	26.00	10.1038/s41467-023-41005-2
ZnN_2H_8	0.73	15.99	2.29	0.01	10.1063/5.0127365
Zr_3AlH	0.20	478.25	1.57	2.00	10.1103/PhysRevLett.132.166001

TABLE S3. List of quaternary hydrides at ambient pressure.

**Name**	H_f	M_X/M_H	χ	T_c **(K)**	**Ref.**
K_2LiCuH_6	0.60	24.58	1.77	47.10	10.1002/adfm.202404043
Na_2LiCuH_6	0.60	19.26	1.79	45.90	10.1002/adfm.202404043
Na_2MgCuH_6	0.60	22.13	1.83	24.70	10.1002/adfm.202404043
K_2InCuH_6	0.60	42.42	1.85	53.00	10.1002/adfm.202404043
Na_2CdCuH_6	0.60	36.70	1.87	26.00	10.1002/adfm.202404043
Li_2CuGaH_6	0.60	24.33	1.89	37.90	10.1002/adfm.202404043
Pb_2CuRuH_6	0.60	95.74	2.20	23.70	10.1002/adfm.202404043
Na_2GaRuH_6	0.60	35.84	1.91	35.20	10.1002/adfm.202404043
Na_2SiPdH_6	0.60	29.84	1.92	39.80	10.1002/adfm.202404043
Mg_2SrPdH_8	0.67	30.09	1.95	49.10	10.1002/adfm.202404043
Mg_2BaPdH_8	0.67	36.25	1.94	41.60	10.1002/adfm.202404043
Cs_2NaSnH_6	0.60	67.38	1.77	34.50	10.1002/adfm.202404043
Na_2PdIrH_6	0.60	56.98	1.95	23.50	10.1002/adfm.202404043
$CsBePtH_6$	0.67	55.72	1.98	30.90	10.1002/adfm.202404043
$KBePtH_6$	0.67	40.21	1.99	39.90	10.1002/adfm.202404043
$RbBePtH_6$	0.67	47.88	1.99	37.90	10.1002/adfm.202404043
Mg_2SrPtH_8	0.67	41.09	1.95	49.40	10.1002/adfm.202404043
Mg_2BaPtH_8	0.67	47.25	1.95	38.40	10.1002/adfm.202404043
K_2HgAuH_6	0.60	78.66	1.91	32.50	10.1002/adfm.202404043
Na_2LiZnH_6	0.60	19.56	1.77	37.80	10.1002/adfm.202404043
K_2AlCdH_6	0.60	35.98	1.81	21.00	10.1002/adfm.202404043
K_2AlHgH_6	0.60	50.56	1.85	32.50	10.1002/adfm.202404043
Rb_2AlHgH_6	0.60	65.89	1.85	31.40	10.1002/adfm.202404043
K_2InAgH_6	0.60	49.75	1.86	31.70	10.1002/adfm.202404043
K_2AgCdH_6	0.60	49.35	1.85	27.50	10.1002/adfm.202404043

3. List of quaternary hydrides reported in the literature with $H_f \geq 0.8$

TABLE S4. Quaternary hydrides reported in previous studies that exhibit high hydrogen content ($H_f \geq 0.8$) and descriptor values characteristic of high-T_c superconducting candidates.

**Name**	**Space group**	H_f	M_X/M_H	χ	p **(GPa)**	T_c **(K)**	**Ref.**
$LaYBe_2H_{16}$	P4/mmm	0.80	15.24	2.03	75	221.00	[4]
$Ba2SiPH_{16}$	P4/mmm	0.80	20.69	2.05	60	160.00	[4]
$AcLa_3Be_4H_{32}$	$Pm\bar{3}m$	0.80	21.07	2.03	20	153.18	[5]
$LaSrB_2H_{16}$	P4/mmm	0.80	15.16	2.02	55	143.00	[4]
$AcBaSi_2H_{16}$	P4/mmm	0.80	26.07	2.05	30	137.00	[4]
$LaAc_3Be_4H_{32}$	$Pm\bar{3}m$	0.80	26.54	2.03	20	134.94	[5]
$ThLa_3Be_4H_{32}$	$Pm\bar{3}m$	0.80	21.23	2.03	10	134.33	[5]
$ThLa_3Be_4H_{32}$	$Pm\bar{3}m$	0.80	21.23	2.03	20	129.94	[5]
$LaThBe_2H_{16}$	P4/mmm	0.80	24.12	2.04	10	126.00	[4]
$CaTh_3Be_4H_{32}$	$Pm\bar{3}m$	0.80	23.94	2.04	5	123.96	[5]
$AcSrSi_2H_{16}$	P4/mmm	0.80	22.99	2.05	30	123.00	[4]
$ThAc_3Be_4H_{32}$	$Pm\bar{3}m$	0.80	29.42	2.03	20	119.15	[5]
$CaTh_3Be_4H_{32}$	$Pm\bar{3}m$	0.80	23.94	2.04	15	118.93	[5]
$CaTh_3Be_4H_{32}$	$Pm\bar{3}m$	0.80	23.94	2.04	20	114.60	[5]
$ThAc_3Be_4H_{32}$	$Pm\bar{3}m$	0.80	29.42	2.03	5	113.41	[5]
$SrTh_3Be_4H_{32}$	$Pm\bar{3}m$	0.80	25.42	2.04	5	112.29	[5]
$SrTh_3Be_4H_{32}$	$Pm\bar{3}m$	0.80	25.42	2.04	20	107.85	[5]
$YCeBe_2H_{16}$	P4/mmm	0.80	15.32	2.03	45	104.00	[4]
$BaTh_3Be_4H_{32}$	$Pm\bar{3}m$	0.80	26.96	2.04	10	101.67	[5]
$BaTh_3Be_4H_{32}$	$Pm\bar{3}m$	0.80	26.96	2.04	20	99.50	[5]
$AcTh_3Be_4H_{32}$	$Pm\bar{3}m$	0.80	29.74	2.04	10	79.11	[5]
$LaTh_3Be_4H_{32}$	$Pm\bar{3}m$	0.80	27.01	2.04	5	78.12	[5]
$LaTh_3Be_4H_{32}$	$Pm\bar{3}m$	0.80	27.01	2.04	20	77.39	[5]
$AcTh_3Be_4H_{32}$	$Pm\bar{3}m$	0.80	29.74	2.04	20	74.98	[5]
$LaCeBe_2H_{16}$	P4/mmm	0.80	18.42	2.03	20	68.00	[4]

4. Filtering criteria vs total number of generated compounds

TABLE S5. Set of descriptor parameters and the resulting total number of obtained compounds. For general formula of $A_\alpha B_\beta C_\gamma H_\delta$ the stoichiometric coefficients were set to $\alpha, \beta, \gamma \in \{1, 2, 3\}$ and $\delta \in \{2, 4, 6, 8, 9, 10, 12, 16, 20, 24, 28, 32, 36\}$.

**Set of parameters**	**Total number of compounds**
$\chi \in [2.0, 2.1]$, $H_f \geq 0.8$, $M_X/M_H \leq 20$	1,060,019
$\chi \in [1.9, 2.2]$, $H_f \geq 0.8$, $M_X/M_H \leq 20$	3,813,319
$\chi \in [2.0, 2.1]$, $H_f \geq 0.8$, $M_X/M_H \leq 40$	3,829,167
$\chi \in [1.9, 2.2]$, $H_f \geq 0.8$, $M_X/M_H \leq 40$	9,631,312

TABLE S6. Set of descriptor parameters and the resulting total number of obtained compounds. For general formula of $A_\alpha B_\beta C_\gamma H_\delta$ the stoichiometric coefficients were set to $\alpha, \beta, \gamma \in \{1, 2, 3, 4\}$ and $\delta \in \{2, 4, 6, 8, 9, 10, 12, 16, 20, 24, 28, 32, 36\}$.

**Set of parameters**	**Total number of compounds**
$\chi \in [2.0, 2.1]$, $H_f \geq 0.8$, $M_X/M_H \leq 20$	1,583,630
$\chi \in [1.9, 2.2]$, $H_f \geq 0.8$, $M_X/M_H \leq 20$	5,098,396
$\chi \in [2.0, 2.1]$, $H_f \geq 0.8$, $M_X/M_H \leq 40$	6,041,654
$\chi \in [1.9, 2.2]$, $H_f \geq 0.8$, $M_X/M_H \leq 40$	14,134,979

5. Electronegativity values (Pauling scale) of elements used to create hydrogen compounds

TABLE S7. Electronegativity values (Pauling scale) for elements with well-defined values and stable, non-radioactive isotopes. Noble gases are excluded due to their negligible tendency to form hydrides.

Element	χ	Element	χ	Element	χ	Element	χ
Hydrogen (H)	2.20	Lithium (Li)	0.98	Beryllium (Be)	1.57	Boron (B)	2.04
Carbon (C)	2.55	Nitrogen (N)	3.04	Oxygen (O)	3.44	Fluorine (F)	3.98
Sodium (Na)	0.93	Magnesium (Mg)	1.31	Aluminium (Al)	1.61	Silicon (Si)	1.90
Phosphorus (P)	2.19	Sulfur (S)	2.58	Chlorine (Cl)	3.16	Potassium (K)	0.82
Calcium (Ca)	1.00	Scandium (Sc)	1.36	Titanium (Ti)	1.54	Vanadium (V)	1.63
Chromium (Cr)	1.66	Manganese (Mn)	1.55	Iron (Fe)	1.83	Cobalt (Co)	1.88
Nickel (Ni)	1.91	Copper (Cu)	1.90	Zinc (Zn)	1.65	Gallium (Ga)	1.81
Germanium (Ge)	2.01	Arsenic (As)	2.18	Selenium (Se)	2.55	Bromine (Br)	2.96
Rubidium (Rb)	0.82	Strontium (Sr)	0.95	Yttrium (Y)	1.22	Zirconium (Zr)	1.33
Niobium (Nb)	1.60	Molybdenum (Mo)	2.16	Ruthenium (Ru)	2.20	Rhodium (Rh)	2.28
Palladium (Pd)	2.20	Silver (Ag)	1.93	Cadmium (Cd)	1.69	Indium (In)	1.78
Tin (Sn)	1.96	Antimony (Sb)	2.05	Iodine (I)	2.66	Cesium (Cs)	0.79
Barium (Ba)	0.89	Lanthanum (La)	1.10	Cerium (Ce)	1.12	Praseodymium (Pr)	1.13
Neodymium (Nd)	1.14	Samarium (Sm)	1.17	Europium (Eu)	1.20	Gadolinium (Gd)	1.20
Terbium (Tb)	1.10	Dysprosium (Dy)	1.22	Holmium (Ho)	1.23	Erbium (Er)	1.24
Thulium (Tm)	1.25	Ytterbium (Yb)	1.10	Lutetium (Lu)	1.27	Hafnium (Hf)	1.30
Tantalum (Ta)	1.50	Tungsten (W)	2.36	Rhenium (Re)	1.90	Osmium (Os)	2.20
Iridium (Ir)	2.20	Platinum (Pt)	2.28	Gold (Au)	2.54	Mercury (Hg)	2.00
Thallium (Tl)	1.62	Lead (Pb)	2.33	Bismuth (Bi)	2.02	Thorium (Th)	1.30
Protactinium (Pa)	1.50	Uranium (U)	1.38	Neptunium (Np)	1.36	Plutonium (Pu)	1.28
Americium (Am)	1.13	Curium (Cm)	1.28	Berkelium (Bk)	1.30	Californium (Cf)	1.30
Einsteinium (Es)	1.30	Fermium (Fm)	1.30	Mendelevium (Md)	1.30	Nobelium (No)	1.30
Lawrencium (Lr)	1.30						

[1] Chris J. Pickard, Ion Errea, and Mikhail I. Eremets, "Superconducting hydrides under pressure," Annu. Rev. Condens. Matter Phys. **11**, 57–76 (2020).

[2] Izabela A. Wrona, Pawel Niegodajew, and Artur P. Durajski, "A recipe for an effective selection of promising candidates for high-temperature superconductors among binary hydrides," Materials Today Physics **46**, 101499 (2024).

[3] Izabela A. Wrona, Paweł Niegodajew, and Artur P. Durajski, "High-temperature ternary superhydrides: A strategic roadmap to optimal superconducting parameters," Advanced Functional Materials **35**, 2423680 (2025).

[4] Xudong Wei, Xiaokuan Hao, Xiaoxu Song, Jingyu Niu, Guoying Gao, and Yongjun Tian, "Design of high-Tc quaternary hydrides under moderate pressures through substitutional doping," Advanced Functional Materials **35**, 2419457 (2025).

[5] Wendi Zhao, Defang Duan, Decheng An, Qiwen Jiang, Zhengtao Liu, Tiancheng Ma, Zihao Huo, Jianhui Du, and Tian Cui, "High temperature superconductivity of quaternary hydrides XM3Be4H32 (X, M = Ca, Sr, Ba, Y, La, Ac, Th) under moderate pressure," Materials Today Physics **43**, 101387 (2024).
